# Timing errors and temporal uncertainty in clinical databases—A narrative review

**DOI:** 10.3389/fdgth.2022.932599

**Published:** 2022-08-18

**Authors:** Andrew J. Goodwin, Danny Eytan, William Dixon, Sebastian D. Goodfellow, Zakary Doherty, Robert W. Greer, Alistair McEwan, Mark Tracy, Peter C. Laussen, Azadeh Assadi, Mjaye Mazwi

**Affiliations:** ^1^Department of Critical Care Medicine, The Hospital for Sick Children, Toronto, ON, Canada; ^2^School of Biomedical Engineering, University of Sydney, Sydney, NSW, Australia; ^3^Department of Medicine, Technion - Israel Institute of Technology, Haifa, Israel; ^4^Department of Civil and Mineral Engineering, University of Toronto, Toronto, ON, Canada; ^5^Research Fellow, School of Rural Health, Monash University, Melbourne, VIC, Australia; ^6^Neonatal Intensive Care Unit, Westmead Hospital, Sydney, NSW, Australia; ^7^Department of Paediatrics and Child Health, The University of Sydney, Sydney, NSW, Australia; ^8^Department of Anesthesia, Boston Children's Hospital, Boston, MA, United States; ^9^Department of Engineering and Applied Sciences, Institute of Biomedical Engineering, University of Toronto, Toronto, ON, Canada; ^10^Department of Paediatrics, University of Toronto, Toronto, ON, Canada

**Keywords:** time, clocks, uncertainty, clinical, ICU, medicine, errors, metrology

## Abstract

A firm concept of time is essential for establishing causality in a clinical setting. Review of critical incidents and generation of study hypotheses require a robust understanding of the sequence of events but conducting such work can be problematic when timestamps are recorded by independent and unsynchronized clocks. Most clinical models implicitly assume that timestamps have been measured accurately and precisely, but this custom will need to be re-evaluated if our algorithms and models are to make meaningful use of higher frequency physiological data sources. In this narrative review we explore factors that can result in timestamps being erroneously recorded in a clinical setting, with particular focus on systems that may be present in a critical care unit. We discuss how clocks, medical devices, data storage systems, algorithmic effects, human factors, and other external systems may affect the accuracy and precision of recorded timestamps. The concept of temporal uncertainty is introduced, and a holistic approach to timing accuracy, precision, and uncertainty is proposed. This quantitative approach to modeling temporal uncertainty provides a basis to achieve enhanced model generalizability and improved analytical outcomes.

“*A man with a watch knows what time it is. A man with two watches is never sure.”* – Segal's Law

## 1. Introduction

Time is an essential concept in both clinical research and the practice of medicine in general (1–6). This is especially true in areas of practice such as critical care where large amounts of data are being collected, and where accurate and consistently recorded times are essential for the creation of a defensible medical record (7–9). A clear sense of the temporal relationship between an exposure or treatment and the subsequent patient condition is the means by which clinicians generate differential diagnoses and determine the efficacy of treatments (10–13). The significance of time in medical decision-making is underscored by the field's reliance on time series data as a means of determining patient trajectories in longitudinal monitoring (14), for example while following the progression of septic shock (15) or monitoring glucose levels in diabetic patients (16). Recent research focusing on clinical time series data has also demonstrated that actionable information may be embodied in the *timestamps alone*. i.e., the sequence and timing of the data collection process contains information above and beyond the physiological values that are being measured and recorded (17, 18).

Although identification of causal relationships, review of critical incidents, and generation of study hypotheses all require a robust understanding of the sequence of events (19), conducting such work in a clinical setting can be problematic when timestamps are recorded by independent and unsynchronized clocks (20–23). Additionally, the process of transferring timestamps into clinical and research databases may be adversely impacted by a multitude of random errors (24). These errors affect the measurement of time, thereby creating a situation where the timestamps stored in clinical databases may not necessarily represent the true time that an event occurred.

Measurement errors in general will typically occur early in the modeling process. These errors introduce bias and uncertainty that will propagate through downstream analysis (25–27), confounding the process of research and discovery of new phenomena (28–31). While quantification of uncertainty and analysis of measurement errors are ubiquitous concepts in fields such as physics and engineering (32–35) such techniques remain relatively uncommon in the medical literature (36) making it difficult for readers to judge the robustness of clinical research (37–39).

The temporal component of physiological measurements is important, and therefore should also be considered when assessing the impact of measurement errors (40), yet this process has been constrained by the fact that medicine has been relatively slow to embrace a robust approach to the measurement of time (41–43). To address this problem, we need to establish a formal and principled discipline around identification of timing errors and quantification of *temporal uncertainty* (44–46).

### 1.1. Motivation and overview

Our research group is embedded in critical care, a dynamic environment in which the patient condition can change rapidly, and where such changes are associated with significant modification of risk. Since 2016 we have been continuously recording high frequency physiological data streams in the critical care unit at The Hospital for Sick Children in Toronto, Ontario and have amassed a database of over one million patient-hours of data, comprising more than four trillion unique physiological measurements (47). Each observation in this database has been assigned a millisecond precision timestamp by at least two separate clocks, providing an ideal substrate for exploring the behavior of timekeeping systems in the critical care environment (48). As part of our efforts to develop robust physiological models and clinical decision support systems we have concluded it was prudent to explore potential biases and uncertainties around all sources of information used in these models, with an initial focus on the measurement of time (49, 50).

In order to achieve this goal, we began by identifying all timekeeping devices and by noting potential sources of timing error in an attempt to establish a preliminary understanding of their individual and collective impact (51). During this process we considered erroneous timepieces (Section 2), delays due to algorithmic effects (Section 3), human factors (Section 4), and random errors introduced by software and other systems (Section 5).

Building on this foundation we adopted a holistic approach to the measurement of accuracy, precision and uncertainty that could be consistently applied across a wide range of timepieces, data sources, and time scales. We then explored mechanisms by which the accuracy and precision of timestamps in our databases could be improved, either through retrospective correction of systematic errors, or prospectively *via* modification of our data collection procedures and systems (Section 6). Finally, after identifying and correcting systematic timing errors, we quantified any residual temporal uncertainty and incorporated this information as a core component of our physiological modeling and machine learning projects (Section 7).

This narrative review provides a broad overview of the issues we considered during this process, bringing together concepts from metrology, statistics, biomedical engineering, and numerical analysis to introduce the concept of temporal uncertainty in a clinical setting.

### 1.2. Temporal resolution, accuracy, and precision

**Figure 1:**
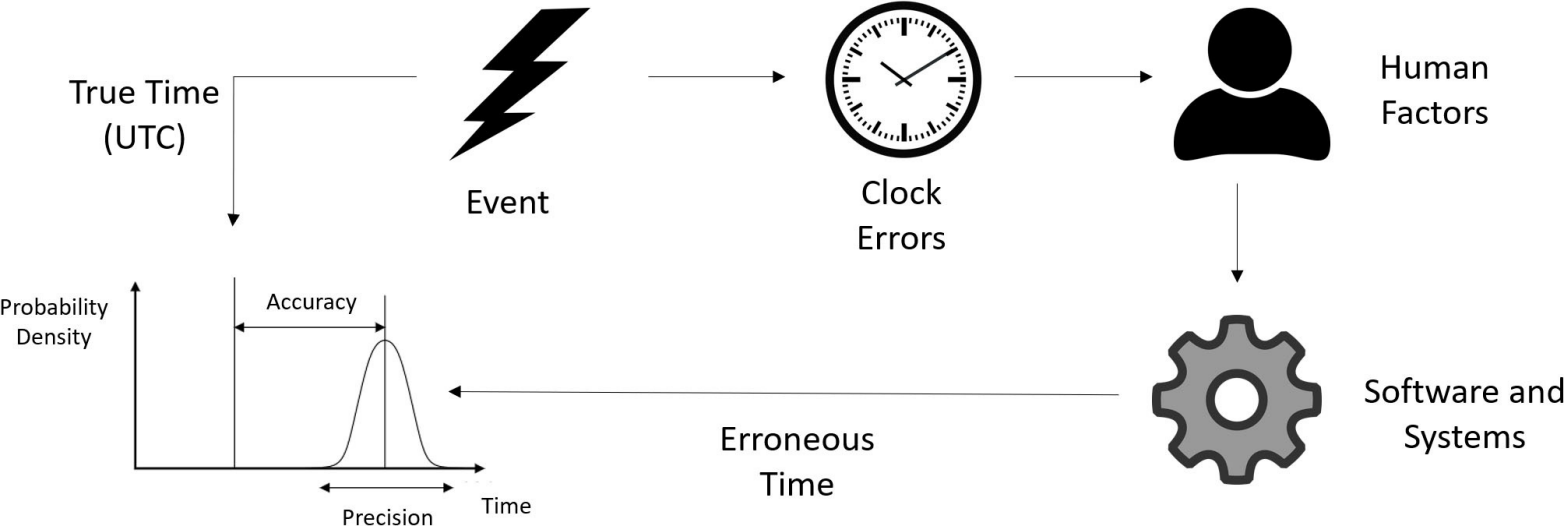
Times recorded in clinical databases may not represent the true time the event occurred. The precision and accuracy of a recorded time may be affected by several different factors.

All measurements are subject to errors (35), including measurements of time (40, 52). We consider the true time and our erroneous measurement of time to be two different variables (53), and we define *accuracy* to be the difference between these two values (see Figure 1). For our purposes we adopted Coordinated Universal Time (UTC) as the underlying, yet unobserved, source of truth (54–56). Where possible we used a relatively accurate and precise master clock as a close approximation of UTC as described in Section 6.1.

We define the *resolution* of a timepiece as its ability to display or discern a precise moment, with lower resolution timepieces being less capable of describing the exact time an event occurred (57). *Precision* is related to resolution but is usually used to describe uncertainty that results from a random process. Modeling uncertainty due to precision and resolution is discussed in detail in Section 7.1. The terms precision and resolution will be used interchangeably throughout this review.

It is important to note that timing errors and uncertainties not only relate to the accuracy and precision of discrete times, but also to the accuracy and precision of the *rate* at which measured times drift away from true time, i.e., we also need to measure the quality of our timestamps *as a function of time* (see Section 2.2).

### 1.3. Temporal uncertainty

As an illustration of the utility of temporal uncertainty, consider as an example the situation where someone says “I'll meet you at the store in twenty minutes” and how that statement feels different from someone saying “I'll meet you at the store in *nineteen* minutes”. The presence of a rounded number in the former statement conveys a sense of indeterminacy about the time period, whereas “nineteen minutes” may be understood by a human listener as being somewhat more precise. Computer algorithms however are unable to understand this kind of implied precision and will assume that all recorded times are equally precise unless more information is explicitly provided. Our goal with this work is to quantify the uncertainty associated with measurements of time, and to make this information explicitly available to our models as an additional property of each temporal measurement.

Uncertainties in general may be classified as either *epistemic* or *aleatoric*, where epistemic uncertainties may be modeled and reduced, and aleatoric uncertainties may be characterized but not reduced (58, 59). It is useful to keep these distinctions in mind since our goal in this work is to identify, model, and correct epistemic temporal uncertainties, and to represent any remaining aleatoric temporal uncertainty as a probability density function. Techniques that can be used to model temporal uncertainty are discussed in Section 7.

Aleatoric uncertainties may be further categorized into *homoscedastic* uncertainty, where the variance of the errors remains constant for all recorded times, and *heteroscedastic* uncertainty, where some measurements of time in the same dataset are noisier than others (60). Situations that result in heteroscedastic temporal uncertainties are discussed in Section 7.2.

## 2. Erroneous timepieces in a clinical environment

Complex clinical environments may contain multiple devices that are all capable of recording timestamped data. If these devices are not synchronized it can be difficult to know which clock (if any) was the source of truth when retrospectively analyzing data. In this section we summarize the work of other groups who have observed inaccurate clocks in a clinical setting, and we describe the mechanisms that result in erroneous measurement of time.

### 2.1. Inaccurate clocks

Medical devices such as ventilators, cardiac monitors, regional saturation monitors, and renal replacement therapy devices may all individually use different approaches to how they manage and report time (61). Some devices may use a proprietary time synchronization system, while others may keep time independently, requiring manual programming to ensure that the displayed time is accurate (22, 62, 63). As a result of this variability, a single event observed by different medical devices may be recorded with different timestamps. Similarly, the elapsed time between two related events may be erroneously measured if different devices are used to record each event.

The accuracy of clocks in clinical settings has been documented by many researchers, including one study by Goldman who examined over 1700 clocks and over 1300 medical devices at four different hospitals (64). This investigation found that inaccurate timekeeping was pervasive and concluded that significant cost savings and improvements to patient safety could be gained if more rigorous approaches to timekeeping were adopted. Other groups have also published evidence of clinical timekeeping issues (4, 62, 65–76), and the potential hazards associated with unsynchronized clocks in medical devices was considered to be so serious by the Pennsylvania Patient Safety Authority that they issued an advisory warning about the problem in 2012 (77).

**Figure 2:**
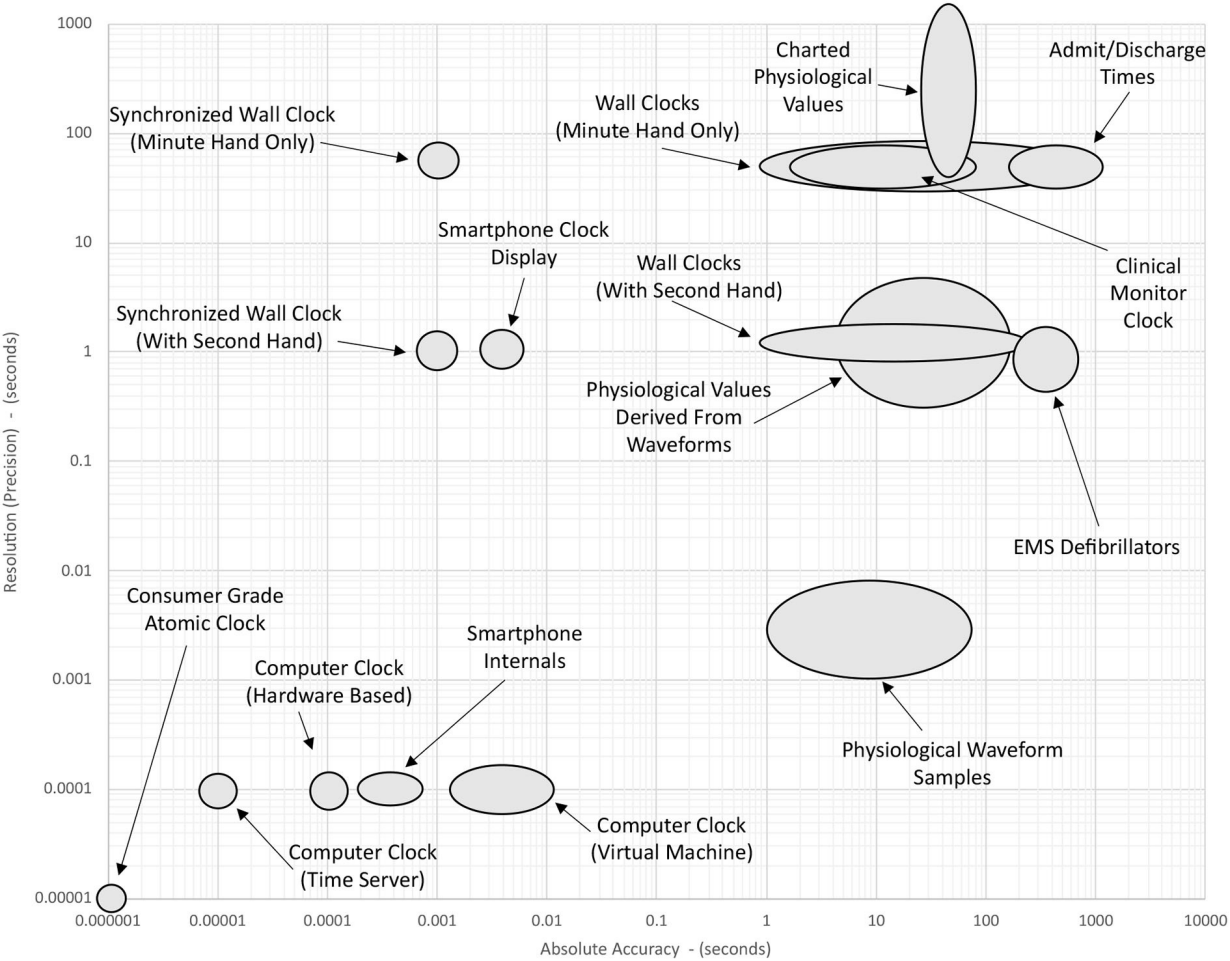
Approximate accuracy and resolution of different time sources in the ICU. Positions of the ellipses are determined by the summation of factors discussed in the literature cited in Sections 1–4. The position of a device shown in this figure indicates its approximate precision and accuracy *before* any of the timekeeping improvements described in Section 6 have been implemented.

Figure 2 gives a schematic overview of the accuracy and resolution of various sources of time that may be found in a typical intensive care environment. The data shown in this figure are conceptual estimates only and have been generated by collating information from the research cited in Sections 2–5. Note that each axis has a logarithmic scale that spans eight orders of magnitude. Elongated ellipses are used to indicate that the precision or accuracy of a particular timepiece may be more variable along one of the axes. The position of a device shown in this figure indicates its approximate precision and accuracy *before* any of the timekeeping improvements described in Section 6 have been implemented.

### 2.2. Clock drift

Drift is defined as the gradual change in the magnitude of the measurement error over time (78). In the case of timing errors, drift results from a situation where a timepiece does not run at exactly the same rate as an accurate reference clock. The units of clock drift are *seconds* (of error) *per second* which is often stated as *parts per million* (ppm).

The drift rate of clocks and other timekeeping systems can be experimentally measured and systematically corrected. For example, Singh et al. measured the drift rate between clocks in a video camera and in the server housing the Electronic Medical Record (EMR) as part of a study examining the physical manipulation of neonates (79). In this situation, the two timepieces were found to have a relative drift rate of around 140ppm (around 0.5 s per hour).

Even within a seemingly homogeneous system, crystal oscillators and microelectromechanical oscillators may exhibit some variance in frequency (80, 81). The oscillation frequencies of these components may be affected by environmental factors such as variations in temperature or the flexible power management provided by modern microprocessors (82). Vilhar and Depolli encountered this phenomenon when they attempted to synthesize a 12-lead electrocardiogram (ECG) using multiple identical wireless sensors, each of which were observed to have a slightly different sample rate (83). Minor variations in the ECG sample rates of each sensor made it difficult to precisely synchronize the data streams during later analysis (84). Uncertain and variable delays over the wireless network also confounded the problem in this case.

### 2.3. Inaccurate and imprecise sample rates

Precise timing and synchronization of signals requires that the sample rates of sensors are precisely specified, yet medical device manufacturers' advertised sample rates may be inaccurate and should not be relied upon (85–87). These devices typically contain a crystal or microelectromechanical oscillator that generates a very stable resonance frequency which can be used to measure the passage of time. Although the sample rates of these components are often known by the manufacturer to five or more significant figures (80) they may be colloquially reported in documentation and marketing material using more rounded values (88).

For example, Jarchi and Casson found that PPG and motion data recorded using a Shimmer 3 GSR+ unit (Shimmer Sensing, Dublin, Ireland) (89) had a true sample rate of 255.69 Hz despite being marketed as having a sample rate of 256 Hz (90). Similarly, Vollmer et al. measured the true sample frequency of five different wearable biomedical sensors and found that their sample rates differed from the manufacturers stated sample rate by up to 290ppm (87).

Signals with imprecisely defined sample frequencies can become problematic if they are subsequently used for timekeeping purposes (84, 91). This kind of error is especially impactful in digital storage systems that use *inferred time* (see Section 2.4).

### 2.4. Inferred time

Physiological waveforms and other high frequency signals are often stored in a format that does not explicitly define the time for each individual sample, but instead relies on the sample rate to *infer* time. For example, the Waveform Database (WFDB) file format used by Physionet (92) calculates the time as a function of the sample frequency and the number of samples that have elapsed since the start of the file (93). Similarly, the Critical Care Data Exchange Format (94) supports inferred time for the storage of waveform data among several other options (95). Time series data that relies on inferred timestamps may be susceptible to drift as a result of imprecisely defined sample rates, and uncertainty associated with this imprecision will be cumulative. This concept is discussed in more detail in Section 7.1.

## 3. Algorithmic effects

A typical pre-processing pipeline for time series data may involve multiple computational steps (96, 97), each of which has the potential to impact timestamps in some way. These algorithms may either be applied to the data within a medical device, or may be the result of external data processing, or both (98). In this section, we discuss various algorithms that may be applied to physiological time series data and how these algorithms can introduce delays into the timestamps.

### 3.1. Moving averages and temporal abstraction

Multimodal medical time series will often comprise one or more irregularly sampled data streams (99–101). These data may be subsequently altered by parameterization or regularization of time, generating a periodic sequence that is smoother, more continuous, and therefore more amenable to analysis (102–104). Care must be taken however when calculating a moving average as several methods are available, each of which may affect the timing of the resultant data in different ways (105).

The Philips X2 multi-module, for instance, calculates the heart rate during normal rhythm by taking the average from the last twelve R-R intervals (106). Similarly, pulse oximetry is typically calculated as a moving average, introducing a delay with a magnitude related to the size of the averaging window (107–109).

### 3.2. Pre-processing of physiological waveforms

High-frequency physiological waveforms may be subject to a wide variety of smoothing, filtering, and buffering processes, each of which can introduce delays of different magnitudes. When two signals experience a different delay before being timestamped then they may appear to be out of phase when subsequently analyzed. Offsets of this kind are mentioned in the documentation for the MIMIC-II database, which informs users that the signals are not suitable for inter-waveform analysis (110). Sukiennik et al. overcame this problem by quantifying and correcting the delay between ECG and Arterial Blood Pressure (ABP) waveforms before calculating a cross correlation of the two signals (111). Variable delays were also documented in a different context by Bracco and Backman who observed that the display of the ABP waveform on a patient monitor was delayed by 900 ms, whereas the display of the photoplethysmography (PPG) waveform on the same monitor was delayed by 1,400 ms (112).

Adding complexity to this issue is the fact that the magnitude of the delay on an individual signal may not be constant with respect to time (113). Several groups have recently explored this phenomenon in PPG waveforms where the delay was observed to follow a characteristic saw-tooth pattern over time (114, 115).

### 3.3. Variables derived from physiological waveforms

Although physiological waveforms are potentially a rich source of insight into patient condition, they are cumbersome and typically not stored permanently in the EMR (11, 47). Instead, lower frequency derivative values are generated that describe the trend in behavior of the underlying signal (116). Classic examples of this include the calculation of Heart Rate (HR) from R-peaks in a patient's ECG (117) and derivation of diastolic and systolic blood pressures from the peaks and troughs of the ABP waveform (118). Algorithms that derive such features from physiological waveforms often introduce a delay in the availability of the derivative value, and the magnitude of the delays between derived variables may differ (119–121).

## 4. Human factors

Clinical time series data can incorporate events that were not automatically recorded by clinical monitors (122, 123). This aspect of the data collection process may be impacted by the biases, fallibility, and unpredictability of the person recording the data. Examples of such information include the timing and dosage of therapeutic drugs, times of blood sample extraction, or any other form of data relying on handwritten notes. This section describes how these human factors can introduce errors into the measurement of time.

### 4.1. Access to multiple sources of time

**Figure 3:**
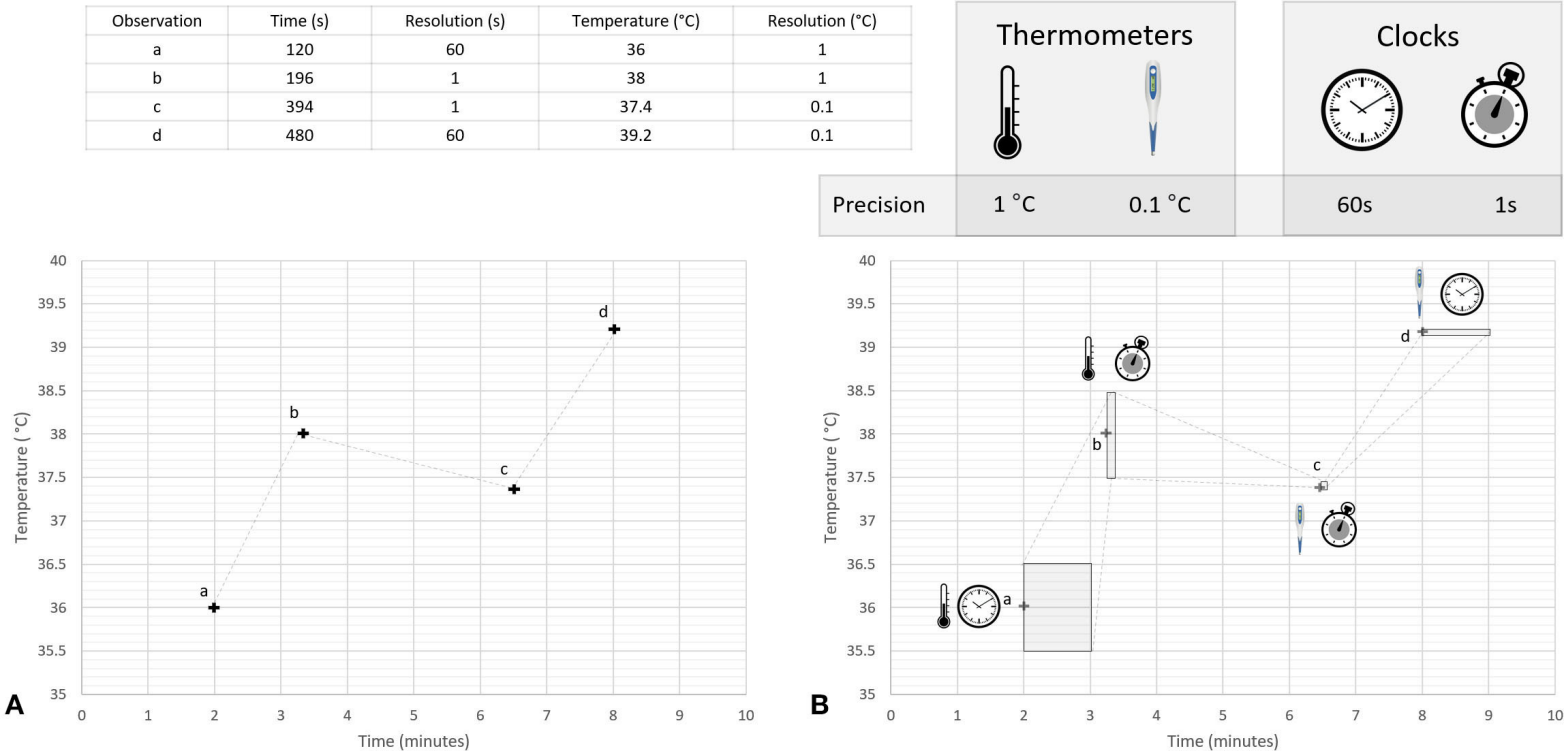
Conceptual illustration of heteroscedastic uncertainties associated with a time series of temperature measurements. This figure illustrates concepts discussed in the literature cited in Sections 4.3, 7.1, and 7.2. Two different thermometers were used to measure temperatures during a hypothetical experiment, a mercury thermometer with a resolution of 1°C and a digital thermometer with a resolution of 0.1°C; times for each temperature were recorded using two different timepieces, a wall clock with a temporal resolution of 1 min and a wristwatch with temporal resolution of 1 s. Four observations (labeled a, b, c, and d) are made using different combinations of these clocks and thermometers. Panel **(A)** shows a plot of the numerical values displayed on the instruments when making the observation, while Panel **(B)** uses shaded regions to represent the range of possible true values that could have resulted in these values. The shape of the shaded regions in Panel **(B)** are determined by the temporal and thermal resolution of the instruments used to make each individual observation. For simplicity, other sources of uncertainty are not considered in this figure.

Multiple timepieces may be within view at any given moment, each of which may be displaying a different time (124). Researchers or clinicians will sometimes randomly select which of these timepieces to use when making an observation. This effect was demonstrated by Ferguson et al. who found significant variability in timepiece preferences among clinicians during emergencies, with 50% of respondents relying on wall clocks, and 46% electing to use personal timepieces (22). Observations made using randomly selected timepieces will contain a mix of errors stemming from the accuracy of the available clocks. Even in a well-synchronized system the intrinsic resolution of each available timepiece may differ, resulting in a mix of aleatoric uncertainties of different magnitudes being associated with the measurements. This concept is discussed in more detail in Section 7.1 and is illustrated in Figure 3.

### 4.2. Human perception of elapsed time

Human perception of time is fallible (125), and accurate recollection of elapsed time may be impacted by exposure to stressful situations (126–130). The expectation that bedside providers deliver both direct care *and* simultaneously record the details of that care creates a need to chart interventions retrospectively (131, 132), potentially requiring recall to determine exactly what time they occurred (133, 134). This problem is exacerbated by the fact that the number and complexity of patient care related tasks increases with patient acuity (104, 135).

### 4.3. Rounding of times

Times may be arbitrarily or systematically rounded to a nearby integer value during the recording or transcription process (136). In one study, 7% of intubations after cardiac arrest were found to have been erroneously recorded as having occurred after 0 min had elapsed since the arrest (137). The authors later explained that all time intervals had been rounded down to the nearest minute, thereby reducing their duration by up to 60 s (138).

Times may also be rounded to simplify a decision-making process, either as part of a formal rule-based model (139), or as a mental shortcut used to streamline the calculation of probabilities when assessing risk (135, 140). The act of rounding a timestamp decreases its precision, which in turn increases its aleatoric uncertainty. Modeling uncertainty due to imposed precision is discussed in more detail in Section 7.1.

### 4.4. Digit preferencing

Spoken and recorded times may be rounded to multiples of 5, 10, 15, 30, or 60 min in a process that is often referred to as *digit preferencing* (141). Rounding to larger multiples of minutes may be done deliberately to convey an increasing sense of imprecision to a human listener, but this connotation is lost once the information is recorded into a database. The prevalence of digit preferencing may increase when event times are retrospectively estimated, or when the *nominal* time of an event is recorded (142).

Locker et al. observed a wide variety of digit preference patterns in admission and discharge times from 137 emergency departments in England and Wales (143). Unsurprisingly the study concluded that rounded times were more prevalent in departments where manual systems of recording were used. This phenomenon has also been explored by several other groups in relation to emergency department admission and discharge times (144, 145), anesthesia start and end times (146, 147), and during the documentation of cardiac arrests (148).

Digit preferencing is similar to rounding in the sense that quantization of the minute component of the timestamp decreases its temporal resolution. When manually rounding to the nearest 5 min then, at least conceptually, the effect is the same as if the time had been accurately read from a clock that has a *resolution* of 5 min. In cases involving a mixture of different rounding options (i.e., a combination of rounding to the nearest 5, 10, 15, 30 min etc.) the resulting mix of resolutions is effectively dependent on a series of random decisions made during the data collection process (149). Datasets that contain a mixture of different temporal resolutions are discussed in more detail in Section 7.2.

### 4.5. Transcription errors

Timing errors can be introduced if text is misread or mistyped while it is being transcribed from one location to another (64, 150, 151). These errors, known as *transcription errors*, are surprisingly common and can result in the introduction of random errors or corruption of timestamps (152). Transcription errors may occur more frequently during periods when large numbers of events are recorded over a short period of time, or when observations are made in the heat of the moment (153, 154).

## 5. Impact of software, networks, and systems

A complex clinical environment, such as an intensive care unit, can contain a myriad of different devices and systems (155). These systems may function in a way that delays, shifts, or otherwise alters the timestamps associated with the data. In this section, we describe how the operation (and *inter*-operation) of these systems can impact the accuracy and precision of temporal measurements. Note that these kinds of errors can be difficult to model as they are often the result of random processes.

### 5.1. Device integration systems and streaming data

Medical devices and other clinical systems will often re-sample or buffer digital signals before releasing them to downstream devices (156, 157). For example, Burmeister et al. measured a buffer-related delay of 30 ms between capture of video and subsequent timestamping inside a video camera system while attempting to synchronize biosignals in their experimental setup (158). Delays of up to 5 s in ECG and 8 s in PPG waveforms were reported by Potera, who also noted that the magnitude of the delay varied with the amount of wireless interference, network load, and server processing time (159, 160).

Medical devices and data integration middleware may also introduce undisclosed latencies (161) or frequent minor adjustments of timestamps to reconcile the differences between the sensor's nominal and true sample rate (90). For example, Charlton found that physiological waveform data acquired using BedMaster software (Excel Medical Electronics, Jupiter, Florida) was sometimes incorrectly timestamped, and suggested some post processing techniques that might be applied to correct the problem (162).

### 5.2. Time shifting and data anonymization

Clinical data will often need to be anonymized before they can be used for research purposes (163). A common method of anonymization involves introduction of random temporal shifts on a per patient basis (164, 165). This technique has been applied to timestamps in the MIMIC-III database for example, where dates have been shifted into the future by a random offset for each individual patient. Although the magnitude of the time shifts applied to the MIMIC database were random, they were performed in a manner that preserved the time of day, day of the week, and approximate seasonality of the original data (166).

Despite the common use of such anonymization approaches, a tension exists between maintaining temporal consistency and preserving privacy (167, 168) as time shifting may remove temporal relationships between signals or disconnect the data from important contextual information (18, 168, 169). Shifting each patient's data by a different random amount may also hinder the ability to perform some kinds of analysis, such as examining the impact of bottlenecks of patients waiting for care on a busy day (170).

### 5.3. Time zones and daylight saving

Time zone settings must be correctly configured in databases to ensure that numerical timestamps are correctly converted into human readable dates and times (171). Incorrectly configured time zone information may also affect the functionality of some clinical algorithms. For example, basal and bolus dosing on insulin pumps have been shown to be impacted by issues relating to daylight saving (172) and when these devices are carried across time zones (173).

Clocks may need to be manually changed at daylight saving changeovers, a process that can take some time to execute (64). The exact time that each clock was changed may be unknown, leading to uncertainty around times recorded during the hours that follow the start or end of daylight saving. Additionally, the schedule of daylight saving transitions may be altered, with changes announced too late to allow software updates to be developed and deployed (174–176).

### 5.4. Lack of standardized definitions

Variability in standards and definitions can lead to inconsistencies in the documentation of clinical events. For example, the definitions of patient length of stay (177, 178), ventilator free days (179, 180) or time of onset of sepsis (181) may vary between different hospitals and jurisdictions. This variability can lead to later confusion about which definition was used for a particular event, making it difficult to compare results across different studies (182).

### 5.5. Software bugs

A wide variety of bugs and formatting issues can arise in relation to management of time in digital systems. For instance, missing times may be represented with a zero, which may in turn be interpreted as midnight by some algorithms (183). Software bugs may introduce random errors that may be detected using temporal conformance checks as discussed in Section 6.5.

## 6. Improving timing accuracy and precision

In this section, we discuss some of the approaches that can be used to reduce timing errors and minimize temporal uncertainties in a clinical environment. A summary of the solutions discussed in this review is presented in Table 1.

**Table 1:** Sources of timing errors and solutions.

**Error type**	**Sections**	**Solution**	**Sections**
Drifting clocks	2.1	Clock synchronization, modeling clock drift	6.1
Delays due to digital filters	3.2	Audit algorithmic delays	6.1
Lag of variables derived from waveforms	3.3, 3.1	Reverse engineer averaging windows	6.2
Imprecisely defined sample frequencies	2.4	Accurately measure all sample frequencies	2.3, 2.4
Lack of standard definitions	5.4	Establish standard nomenclature	5.4
Changes in data collection systems	6.3	Establish “epochs” of data uncertainty	7.2
Transcription errors	4.5	Implement temporal logic checks	6.5
Limitations of digital data types	6.6	Select appropriate temporal data types	6.6
Uncertainty due to rounding of times	4.3	Monte Carlo Analysis	7.1, 7.3
Digit Preferencing	4.4	De-convolve mixed precision datasets	7.2
Access to multiple, inaccurate sources of time	4.1	Clock synchronization, use of a “master clock”	6.1
Fallible human perception of elapsed time	4.2	Estimate extent of possible errors	4.2
Software bugs	5.5	Audit timestamps	6.5
Quantization of recorded times	7.1	Audit resolution of all recorded times	7.1
Unsynchronized signals	6.2	Real-time or retrospective synchronization	6.2
Event time not recorded	N/A	Algorithmically determine event time	6.4

*The solutions generally aim to identify, model, and correct epistemic temporal uncertainties, and to represent any remaining aleatoric temporal uncertainty as a probability density function*.

### 6.1. Choosing a common source of truth for time

Ideally all clinical systems should refer to a master clock as a common source of time (184). This master clock should be both accurate *and* precise, and should also have the ability to provide timestamps programmatically to other systems on a network. If such a time source is unavailable or impractical to use, such as while taking handwritten notes during a resuscitation for example, then a single source of time should be selected and agreed upon by all participants (153).

All other clocks will need to be regularly synchronized with the master clock, either manually or *via* automated synchronization using the Network Time Protocol (NTP) (185–187). NTP can, in principle, be used to synchronize clocks and other medical devices on a network with microsecond precision (188, 189). In practice however, many medical devices lack this capability (62).

Computer clocks are generally well-synchronized and have relatively low drift rates, rendering them suitable as a source of truth in many situations (190, 191). However, care must be taken when using clocks on virtual servers since their drift rates may be higher than hardware based clocks (192–194). While smartphones and wristwatches are ubiquitous and relatively accurate sources of time (195) they can also act as a source of infection, so their use for timing purposes may be discouraged in some clinical situations (196–198).

**Figure 4:**
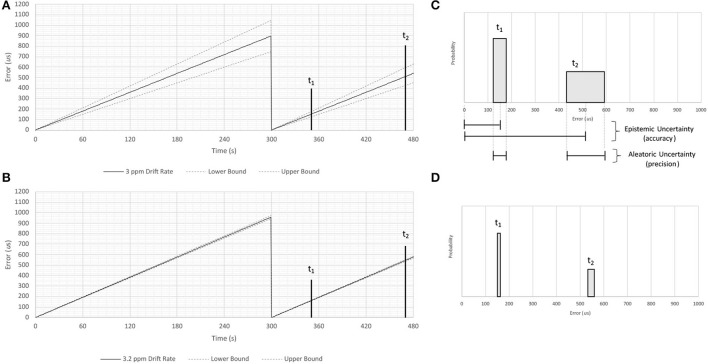
Conceptual illustration of epistemic and aleatoric uncertainties resulting from clock drift. This figure illustrates concepts discussed in the literature cited in Sections 2.2 and 7.1. A series of measurements of time are made using a hypothetical clock that is synchronized with a more accurate timepiece once every 300 seconds. Panel **(A)** shows timing errors caused by a drift rate measured to one significant figure (3 ppm), while panel **(B)** shows timing errors caused by a drift rate measured to two significant figures (3.2 ppm). Shaded regions in Panels **(C,D)** show the accuracy and precision of temporal measurements assuming these two drift rates 50 and 170 s after synchronization (labeled t_1_ and t_2_, respectively). Note that imprecisely specified drift rates result in aleatoric uncertainties that increase as a function of time, and that the magnitude of the uncertainty is inversely proportional to the number of significant figures used to specify the drift rate.

The process of synchronization may cause a clock to “jump” forward or backward in time, creating the appearance of discontinuity in any signal that uses timestamps assigned by that clock (199). Across such a jump a signal will be continuously sampled, but when plotted against the time supplied by the sensor it will appear to have a discontinuity (200). A conceptual example of this phenomenon is illustrated in Figure 4, which shows the reference time of a signal jumping by around 900 μs each time the clock resynchronizes.

### 6.2. Signal synchronization

Precise signal synchronization is required when generating high-fidelity inter-signal correlations (187, 201, 202). Although this capability has been commonplace in other industries for decades (86, 203), bespoke solutions may be required in a clinical environment due to the presence of independent and proprietary timekeeping systems (199).

Physiological signals from different devices may be synchronized *via* simultaneous assignment of timestamps using a highly accurate and independent clock, however this multiplexing approach requires that both signals are routed through a third-party system, a process which may be affected by network jitter and other random effects (204). These variable network delays, and other confounding factors, can be overcome through the use of software such as Lab Streaming Layer (LSL) (205), OpenSynch (206), or corrected retrospectively using algorithmic approaches such as Deep Canonical Correlation Alignment (202). For example, LSL was used by Wang et al. to assess the quality of timing in medical and consumer grade EEG systems (207), and by Siddharth et al. to time align multiple bio-signals including PPG, EEG, eye-gaze headset data, body motion capture, and galvanic skin response (208).

Artifacts or other information common to both signals can be identified and retrospectively aligned in situations where system constraints preclude real-time synchronization. This process may rely upon artifacts that are introduced into the data streams while they are being recorded (201, 209–211), or may utilize features such as heart beats that are naturally present and accessible in a wide range of high frequency physiological signals (212–216). This method of synchronization requires artifacts or features from multiple time points throughout the signals to ensure that clock drift is properly accounted for (85). Another interesting approach to signal synchronization involves simultaneous introduction of white noise to both signals while they are being recorded (217). White noise has rich frequency characteristics that facilitate signal alignment yet is also random enough that it doesn't interfere with the information content of the signals.

Retrospective approaches that rely on common information will only be able align two signals *relative to each other*, i.e., neither signal will necessarily be mapped to UTC time. This may be acceptable in situations where high resolution inter-signal correlation is required but accuracy of the timestamps associated with the derived features is unimportant.

### 6.3. Digitization of data recording systems

Computerized systems can improve timekeeping by eliminating some sources of error such as transcription errors and digit preferencing (146). Deployment of a computerized system will mark the beginning of a new epoch of reduced temporal uncertainty, as both accuracy and precision of recorded times may change after its introduction (218).

Examples of digital timekeeping systems that can be deployed in a clinical setting include computerized EMRs (143, 218), tablet-based applications (219), bar-code systems (220), Radio Frequency Identification (RFID) technology (221), and audio recordings (222).

### 6.4. Algorithmic determination of event times

In some situations, it may be possible to algorithmically determine the times of clinically relevant events *via* the analysis of high frequency physiological data streams. For example, Cao et al. utilized a wide range of signal types to retrospectively determine ventilation times in the MIMIC-II database (223), and Nagaraj et al. determined the times that blood samples were drawn from patients' arterial lines *via* automated detection of associated artifacts in the ABP waveform (224). Information obtained in this manner can be used to augment an incomplete dataset or to improve the temporal accuracy of manually recorded observations.

### 6.5. Temporal conformance checks and auditing

The presence of transcription errors may be revealed *via* implementation of temporal conformance checks (225, 226). These checks will apply logic to search for implausible dates and times in a database. For instance, one study in an Australian drug and alcohol service ran checks on their database and found that 834 out of 9,379 admissions (8.9%) had recorded a start time that was *after* the end time (227). Other examples of temporal conformance checks include ensuring that timestamps from a particular patient encounter fall between the admission and discharge dates (227, 228) and checking that patient age is equal to date of admission minus the date of birth (229). Erroneous timestamps may be flagged and optionally removed before analysis in order to improve the overall temporal accuracy of a dataset, and uncertainties associated with random errors may also be estimated *via* audits designed to assess the integrity of timekeeping activities (230, 231).

### 6.6. Use of appropriate temporal data types

Although it is tempting to use floating point values to represent “continuous” time, these data types can become problematic (91, 232, 233). Not only are such values ultimately quantized in some way (234) but the resolution of floating point data types is dependent on the magnitude of the value being represented (235). Additionally, both floating point and integer data types are unable to exactly represent some frequencies (91, 236). These properties of floating point data types can create problems during numerical analysis, and may result in models that are not reproducible (237). Rounding errors associated with floating point representations of time will accumulate, sometimes with devastating effects (238).

An ideal digital representation of time would be time zone agnostic, contain sufficiently fine resolution to accommodate any analytical technique for which it may be used, and have a range that is large enough to accommodate the timescale being recorded or modeled (239). The smallest unit of time that can be represented in a digital data type will determine its temporal resolution. This smallest unit, referred to as a *chronon* (240), should have a resolution that remains constant throughout the full range of possible values. Longer intervals of time (milliseconds, seconds, hours, etc.) can then be represented by aggregating chronons (241).

## 7. Modeling temporal uncertainty

Quantification of uncertainty plays a pivotal role in the application and optimization of machine learning techniques (242). The reproducibility of analytical approaches can be improved by explicitly considering sources of uncertainty in the input data (243), and numerical methods can be applied that provide more faithful representations of uncertainty than those provided by classical error bounds (244). Despite the existence of such techniques, many clinical models fail to adopt a strict approach toward handling and propagation of uncertainty, perhaps due to a general misconception that the use of very large training datasets will lead to exact results (38, 245, 246).

In Sections 2–5, we described numerous mechanisms that can lead to times being measured erroneously, yet timestamps are typically incorporated into clinical models without any consideration for measurement errors or temporal uncertainty. In this section we outline some methods that can be used to quantify temporal uncertainty, and we give a brief overview of techniques that can be employed these results to be incorporated into downstream analyses (247).

### 7.1. Resolution of times and frequencies

The resolution of a recorded time is determined by the timepiece used to make the observation. For example, a clock that displays only minutes (i.e., a clock with resolution of 1 min) is unable to provide information about what second an event occurred (248). Measurements made using such a clock can be represented as a uniform probability density function which spans the 60 s after the recorded minute, providing a probabilistic representation of the fact that the displayed time is equally likely to represent any moment during the following 60 s. This approach is illustrated in Figure 3B which shows the uncertainties associated with measurements made using two timepieces, each having a different native resolution.

A similar approach can be used when quantifying uncertainty associated with the resolution of a sample frequency. “Resolution” in this case relates to how precisely the sample rate has been defined, i.e., how many significant figures have been used to describe the sample frequency (40). If a medical device manufacturer reports that a signal's sample frequency is 500 Hz then the associated analysis must account for the fact that the fourth significant digit is unknown, and that the *true* sample frequency could be anywhere between 499.5 Hz and 500.5 Hz (36). This range of *possible* sample frequencies (given the specified sample frequency and number of known significant figures) can also be represented using a uniform probability distribution.

An illustrative example of the relationship between sample frequency resolution and its impact on uncertainty is shown in Figure 4. Note that the aleatoric uncertainty grows with time after the moment of synchronization, and that the range of the uncertainty is inversely proportional to the number of significant figures to which the sample rate is known. This uncertainty will hinder one's ability to model and correct any clock drift that might occur when timestamps are inferred by a signal's sample rate.

### 7.2. Heteroscedastic temporal uncertainty

Clinical information can be collected using a wide variety of different systems and devices. This mix of different systems can result in divergent representation of clinical information across different sites (249). One hospital may use an automated system to record admission times for example, while another may use a paper-based system that is potentially subject to rounding and transcription errors (143). Combining these two databases for use in a multi-site research project would generate a single dataset containing timestamps with a mix of temporal accuracies and precisions (i.e., the dataset would contain heteroscedastic temporal uncertainties). Data collected from a single site may also contain heteroscedastic temporal uncertainties, due to a change in temporal accuracy and resolution after the introduction of a new software system for example (167, 219, 250).

A visual representation of heteroscedastic temporal uncertainties is illustrated in Figure 3. This figure shows a hypothetical time series of temperature readings collected using a variety of clocks and thermometers, each with different temporal and thermal resolutions. Shaded regions in Panel B represent the range of true temperatures and times that could have resulted in the quantized information that was recorded in the database. The shape of these shaded regions is different for each measurement, ie, the dataset contains heteroscedastic uncertainties. For simplicity only uncertainties caused by instrument precision are shown in this figure. It is important to note that while heteroscedastic uncertainty is often modeled as being a function of the magnitude of the measurand, this is unlikely to be the cause when considering temporal measurements.

A dataset that has been subject to digit preferencing will also contain timestamps with a mix of different uncertainties. This concept was introduced in Section 4.4 where we demonstrated that times rounded to larger values will have lower resolution, and therefore larger aleatoric uncertainty. Although it is impossible to retrospectively determine which individual observations had been subject to rounding it is still possible to draw some inferences (251). For example, if the timestamps have potentially been rounded to a mix of 1, 5, 10, and 15 min, then a time ending in :17 cannot have been rounded to a multiple of 5, 10, or 15. Similarly, a time ending in 5 could not have been rounded to a multiple of 10. Rietveld developed an approach that could de-convolve the characteristic heaped distribution of minutes that results from digit preferencing and calculate the uncertainty associated with each individual recorded time (as a function of the minute component of the recorded time) (149).

### 7.3. Monte Carlo simulations

Probability density functions representing temporal uncertainty can be used as input into stochastic methods like Monte Carlo models (252–255). These techniques allow uncertainty to be numerically propagated through models and algorithms. Pretty et al. used this approach in their glucose insulin model to propagate uncertainty resulting from timestamps that had been recorded by hand and rounded to the nearest hour (256), and Ward used a Monte Carlo simulation to model the impact of transcription errors on emergency department performance metrics (257).

At a smaller time scale, algorithms that calculate Heart Rate Variability (HRV) are sensitive to the time of the fiducial point of the R-peak. However, uncertainties associated with these times are rarely considered (258). To overcome this problem, the finite resolution of an R-peak can be represented as a uniform probability density function as discussed in Section 7.1. Monte Carlo simulations can then be used to model the impact of R-R intervals with uncertain durations (259, 260). Temporal uncertainty can be propagated through the calculations, generating a distribution of HRV values that may have been possible given the resolution of the times of the R-peaks. The same approach can be used with other features derived from ECG such as example Q-T intervals (261). These techniques may allow more direct comparison of HRV and other features derived from ECG signals that have been acquired using different clinical devices (262).

### 7.4. Biases resulting from measurement errors

Measurement errors in one or more variables can introduce a bias into both linear and non-linear regression techniques (263, 264). Counterintuitively, the bias introduced by the measurement errors does not reduce to zero as the number of observations approaches infinity. The problem can be resolved by using maximum likelihood estimation, but this requires knowledge of the (relative) variance of the errors on *X* and *Y* (265).

Errors in Variables (EIV) models have been used in clinical research to account for measurement errors in general (266–268) and in some situations to account for uncertainty in timestamps in particular. For example, several researchers have used EIV models to account for uncertainty in self-reported meal times while modeling blood glucose trajectories (269, 270) and to account for the difference between the scheduled and actual time of drug administration when modeling pharmokinetics (142).

## 8. Discussion

In this review we have examined sources of errors and uncertainty in relation to recorded times in clinical databases. We have described how multiple sources of error may be associated with a single measurement, with contributions from human, system, and device related factors. These sources of timing error are not mutually exclusive, and their collective impacts are additive. We have also described sources of heteroscedastic temporal uncertainty, where the amount of uncertainty associated with different timestamps within a single time series may be variable. It is evident that timekeeping errors are distressingly common in clinical databases, and it is our opinion that clinical modeling outcomes will be improved if temporal uncertainty is considered when analyzing time series data (133).

Although, in principle, most timing errors can be reduced or eliminated through system improvements, solutions to some clinical timekeeping errors remain elusive. Medical device manufacturers continue to use proprietary time synchronization systems or publish imprecisely defined sample rates in documentation and marketing material. These practices make it difficult to reduce epistemic uncertainties, hindering one's ability to synchronize signals from disparate devices.

When taking physiological measurements, one must accept that poor repeatability is a result of the high variance that is inherent in biological systems (36). Clocks and time in general however are *not* part of the biological system, and therefore should not be given a free pass when it comes to acceptance of variability in measurement between systems. Indeed, a more metrologic approach to *all* aspects of physiological measurement may be key to generating reproducible analytical results (271–274).

The healthcare industry is undergoing a transformation from closed system medical devices to a fully interconnected digital ecosystem (275). Throughout this process, the demand for “off-label” use of high frequency physiological data streams may result in information from clinical monitors being used in ways that were not envisaged by the manufacturer (276). Seemingly inconsequential differences in hardware and software used to capture data can result in datasets with divergent temporal characteristics. Failure to explicitly account for this variability may hamper development and adoption of generalizable of machine learning models (277–279).

We suggest that the following actions be considered when planning improvements to timekeeping systems in a clinical environment:

- Establish a highly accurate and precise master clock as a reference time source
- Synchronize clocks to the master clock using NTP wherever possible
- Introduce data quality initiatives focusing on human factors, such as raising awareness of the impact of rounding practices
- Adopt computer-based documentation approaches during critical events such as resuscitations
- Note the temporal resolution of all timepieces
- Develop an understanding of the strengths and limitations of data types used for storage of dates and times
- Accurately measure the sample rate of all signals, especially where other systems rely on this sample rate to infer time
- Quantify delays due to software and systems
- Model and correct systemic timing errors
- Critically assess timing systems in new medical devices
- Audit databases to reveal the presence of transcription errors
- Identify and characterize sources of heteroscedastic temporal uncertainty
- Note the beginning and end of different “epochs” of temporal uncertainty
- Adopt a data quality framework that facilitate the quantification and storage of temporal uncertainty
- Employ techniques that incorporate temporal uncertainty into analysis.

The issues discussed in this narrative review highlight an increasing need to understand temporal aspects of signal collection and pre-processing in medical devices (280, 281). Our goal in describing these findings has been to spur discussion and to advocate for consensus means by which the medical research community may approach vulnerabilities around time management. We believe that a more standardized approach to time management would provide substantial benefits to the research community, allowing high precision correlation of signals from disparate devices. Standardization of algorithms used in medical devices is essential (282), and, in the end, it may be the users of medical technologies that demand improved interoperability (283).

## 9. Conclusion

Errors in recorded times may have multiple causes and are disturbingly common in clinical environments. These errors can create uncertainty around the temporal sequence of events, confounding analysis and hindering development of generalizable machine learning models. Datasets collected at different hospitals can exhibit divergent temporal characteristics due to the wide range of medical devices, pre-processing methods, and storage systems that may be in use between sites. To mitigate these issues one should identify, quantify, and correct biases in recorded times, and characterize any residual temporal uncertainty so that it can be incorporated into downstream analysis.

## Author contributions

AG: original concept and scoping, wrote the majority of manuscript, generated the figures, sourcing and reading external references, and formatting. DE: paragraphs on clinical relevance, proofreading, and editing. WD: technical support for computer science related sections, support for sections focusing on algorithms, and data types. SG: proofreading, guidance on initial structure and direction, and contributions to sections about algorithms. ZD: proofreading, contribution to digit preferencing section, and suggestions with clinical relevance of the work. RG: technical support, contributions to sections related to systems, and medical devices. AM: proofreading and scoping and technical direction on hardware related paragraphs. PL: initial problem statement, material support, and proofreading. MT: proofreading. AA: proofreading and clinical relevance. MM: writing contributions to initial draft of manuscript, proofreading, guidance on scope and direction, and contributions to discussion around clinical relevance and applications. All authors contributed to the article and approved the submitted version.

## Conflict of interest

The authors declare that the research was conducted in the absence of any commercial or financial relationships that could be construed as a potential conflict of interest. The reviewer BRC declared a shared affiliation with the author PL to the handling editor at the time of review.

## Publisher's note

All claims expressed in this article are solely those of the authors and do not necessarily represent those of their affiliated organizations, or those of the publisher, the editors and the reviewers. Any product that may be evaluated in this article, or claim that may be made by its manufacturer, is not guaranteed or endorsed by the publisher.
